# Interplay Between Immune Checkpoint Modulators and the Epithelial-to-Mesenchymal Transition Axis in Clear Cell Renal Cell Carcinoma

**DOI:** 10.3390/cancers18142258

**Published:** 2026-07-14

**Authors:** Arpita Poddar, Farah Ahmady-Nield, Revati Sharma, Seemadri Subhadarshini, Mohit Kumar Jolly, Suresh Ramakrishna, Ali Raza, Ravi Shukla, George Kannourakis, Aparna Jayachandran, Prashanth Prithviraj

**Affiliations:** 1 Fiona Elsey Cancer Research Institute, Ballarat, VIC 3350, Australia; arpita@fecri.org.au (A.P.); farah@fecri.org.au (F.A.-N.); revupattani@gmail.com (R.S.); alir335612@gmail.com (A.R.); george@fecri.org.au (G.K.); 2 Institute of Innovation, Science and Sustainability, Federation University, Ballarat, VIC 3350, Australia; 3 Ian Potter NanoBiosensing Facility, NanoBiotechnology Research Laboratory, School of Science, RMIT University, Melbourne, VIC 3000, Australia; ravi.shukla@rmit.edu.au; 4 Department of Surgery, The Royal Melbourne Hospital, The University of Melbourne, Parkville, VIC 3050, Australia; 5 Department of Bioengineering, Indian Institute of Science, Bengaluru 560012, Karnataka, India; seemadri.subhadarshini@well.ox.ac.uk (S.S.); mkjolly@iisc.ac.in (M.K.J.); 6 Nuffield Department of Medicine, University of Oxford, Oxford OX3 7BN, UK; 7 Graduate School of Biomedical Science and Engineering, Hanyang University, Seoul 04763, Republic of Korea; suri28@hanyang.ac.kr; 8 College of Medicine, Hanyang University, Seoul 04763, Republic of Korea; 9 Centre for Advanced Materials and Industrial Chemistry, RMIT University, Melbourne, VIC 3001, Australia; 10 School of Health & Biomedical Science, RMIT University, Melbourne, VIC 3000, Australia

**Keywords:** immune checkpoint inhibitors, epithelial-to-mesenchymal transition, clear cell renal cell carcinoma, LAG3, NT5E, PD-L1, survival, biomarkers, single-cell RNA sequencing, AUC

## Abstract

**Background/Objectives**: Clear cell renal cell carcinoma (ccRCC), the predominant malignant subtype of kidney cancer, is the leading cause of death among renal cell carcinoma patients. Although a subset of ccRCC patients benefit from select immune checkpoint inhibitors (ICIs), prognosis remains poor. While PD-1 and PD-L1 have been extensively studied, the prevalence and distribution of other immune checkpoints (ICs) and their relationship with epithelial-to-mesenchymal transition (EMT) remain poorly characterised. Here, we investigated the interplay between twenty ICs and EMT markers and assessed their combined prognostic relevance in ccRCC patients. **Methods**: Transcriptomic profiling and integrated bioinformatic analyses were performed, including differential expression, correlation analyses, survival analyses, forest plot analyses, ROC curve evaluation, and OncoPrint visualisation, complemented by analysis of single-cell RNA sequencing data, immunohistochemistry, and multiplex secretory IC (LegendPlex) assays. **Results**: Transcriptomic profiling of over 500 ccRCC tumours versus normal kidney tissue revealed dysregulation of ICs, particularly LAG3 and NT5E. Notably, expression of ICs, including LAG3 and NT5E, was associated with poor overall survival in 415 ccRCC patients. ICs that synergised with the EMT phenotype provided improved prognostic discrimination compared to individual ICs. Correlation analyses, single-cell RNA sequencing, and immunohistochemistry demonstrated an association between EMT-associated tumours and expression of LAG3 and NT5E. ROC analysis indicated modest prognostic performance of LAG3 and NT5E. **Conclusions**: Collectively, this study identifies an EMT–IC axis in ccRCC and demonstrates its relevance to tumour biology and patient outcomes, highlighting LAG3 and NT5E as potential prognostic markers and therapeutic targets that warrant further investigation.

## Simple Summary

Clear cell renal cell carcinoma (ccRCC) is the most common and most aggressive form of kidney cancer. While some patients benefit from immune checkpoint inhibitor therapies, overall outcomes remain poor for many. Research has mainly focused on PD-1 and PD-L1, whereas the role of other immune checkpoint molecules and their relationship with epithelial-to-mesenchymal transition (EMT), a process associated with tumour progression, immune escape, and metastasis, remains less well understood. In this study, we found that several immune checkpoints were altered in ccRCC tumours, particularly LAG3 and NT5E, and that higher expression of these genes was associated with poorer survival. Integration of immune checkpoint and EMT markers improved prognostic stratification compared with immune checkpoint markers alone. These findings suggest an EMT–immune checkpoint axis involving LAG3 and NT5E that may contribute to ccRCC progression and could help refine risk stratification and guide future therapeutic investigations.

## 1. Introduction

Kidney diseases constitute a major global public health challenge, contributing substantially to morbidity, mortality, and healthcare burden worldwide [1]. Renal cell carcinoma (RCC), the most common form of kidney cancer, comprises a heterogeneous group of malignancies arising from the renal tubular epithelium and affects nearly 400,000 individuals worldwide each year [2]. Clear cell RCC (ccRCC) represents the most frequent and best-studied RCC histological subtype characterised by clear cytoplasm in cells. ccRCC accounts for approximately 80% of all RCC cases and contributes to over 175,000 deaths each year [3,4]. When diagnosed early, ccRCC patients can be successfully treated with surgical or ablative strategies. However, up to a third of ccRCC patients present with metastases, and a third of ccRCC patients develop metastases during follow up [5]. The 5-year survival rate of ccRCC patients is 50%, while metastatic RCC patients have a 5-year survival rate of 5–10% after diagnosis [6,7]. ccRCC is typically non-responsive to traditional chemotherapeutic drugs and radiation therapy [8]. Considering the pathogenetic role of the Von Hippel Lindau (VHL) protein in ccRCC and the highly vascular nature of this RCC subtype, anti-angiogenic strategies with small-molecule tyrosine kinase inhibitors (TKIs) has become a promising therapeutic approach for the treatment of ccRCC [9]. The following TKI small-molecule inhibitor drugs primarily targeting the vascular endothelial growth factor (VEGF) signalling pathway have been approved for clinical use: sunitinib, sorafenib, axitinib, cabozantinib, lenvatinib, and pazopanib [10]. Mammalian target of rapamycin (mTOR) inhibitor drugs everolimus and temsirolimus have been approved for ccRCC patient treatment [11].

More recently, immunotherapy in the form of immune checkpoint inhibition has dramatically changed the standard of care for ccRCC patients [3,12]. Within the tumour microenvironment (TME), immune checkpoint (IC) interactions are often dysregulated, leading to suppressed T-cell effector functions. Immune checkpoint inhibitors (ICIs) effectively targeting these checkpoint molecules can re-animate the tumour-specific T-cell immunity, leading to more durable responses than TKI therapy alone [3,13]. Treatment with ICIs have been effective as ccRCC is an immunogenic tumour with high numbers of immune cells [3,14,15,16]. The best-characterised ICIs targeting Programmed cell-Death 1 (PD-1), Programmed Death Ligand 1 (PD-L1), and Cytotoxic T-lymphocyte-associated antigen 4 (CTLA4) have been shown to reduce the immune escape of cancer cells and suppress tumour growth. Nivolumab or pembrolizumab (anti-PD-1 blockers) and avelumab (anti-PD-L1 blocker) used as monotherapy or in combination with ipilimumab (anti-CTLA4 blocker) or anti-angiogenic drugs in both first- and second-line treatment settings in ccRCC have shown efficacy and overall survival benefits [17,18,19].

Despite the substantial clinical response of ICIs, their efficacy is generally limited to a subset of cancer patients. To date, ICI monotherapies achieve only a modest overall response rate (ORR) of 21–36% [3]. Combination regimens, including dual ICI blockade or ICI–TKI combinations, have improved the ORR to 34–73%; nevertheless, many patients still do not achieve lasting responses, and those who are unresponsive to ICI-based treatments continue to experience poor clinical outcomes [20,21]. Furthermore, high grade 3 or 4 ICI-related adverse events occur in a significant proportion of patients [18,19]. Anti-tumour responses to ICI therapy vary between cancer types and even among patients with the same histological subtype [22,23]. Tumours initially responsive to ICI therapy eventually progress during treatment due to acquired resistance [24]. PD-L1 expression alone is insufficient as a predictive biomarker in ccRCC [25]. Among emerging IC molecules, LAG3 has attracted considerable interest because of its role in T-cell exhaustion and progressive immune dysfunction in ccRCC, in addition to its therapeutic targeting in combination with PD-1 blockade [12,26].

The cellular reprogramming process called epithelial-to-mesenchymal transition (EMT) is a form of cellular plasticity that underlies ICI resistance and promotes relapse, metastases, and unfavourable patient outcomes [27,28]. EMT is a multistep biological process facilitating the switching of cellular phenotypes. During this process, epithelial cells acquire a mesenchymal phenotype and function [29]. The process of EMT leading to cancer initiation, progression, and acquisition of drug resistance has been implicated in immune evasion of cancer cells across several malignancies, including ccRCC [22,23,28,30]. Importantly, EMT is increasingly recognised as a dynamic spectrum of cellular states rather than a binary epithelial-to-mesenchymal transition, with tumour cells frequently adopting partial or hybrid epithelial–mesenchymal phenotypes [31,32]. Emerging evidence links EMT status with altered expression of IC molecules in ccRCC patients [33,34], suggesting that cellular plasticity within the heterogeneous TME directly shapes anti-tumour immune responses.

This study aimed to investigate the interplay between EMT and IC expression in ccRCC, to identify an EMT–IC axis, assess its prognostic relevance, and identify key ICs with potential as biomarkers and therapeutic targets. We compared the expression of IC molecules in healthy kidney and ccRCC tumour samples and analysed a panel of twenty ICs and EMT-associated genes using datasets from SurvExpress and GEO. Prognostic significance was evaluated using survival analyses, forest plots, and receiver operating characteristic curves. LegendPlex assays were performed to assess changes in secretory ICs before and after ICI treatment in a ccRCC patient. Correlation analyses, single-cell RNA sequencing, and immunohistochemistry were utilised to examine the co-expression and spatial association of EMT-like tumour cells with key ICs, particularly NT5E and LAG3. Collectively, these analyses identify an EMT–IC axis, linking EMT phenotype with IC, and highlight NT5E and LAG3 as candidate prognostic markers and therapeutic targets in ccRCC.

## 2. Materials and Methods

### 2.1. OncoPrint Analysis of Immune Checkpoints Using cBioPortal

OncoPrint from cBioPortal (http://cbioportal.org) (accessed on 3 February 2026) was used to obtain a compact graphical summary of gene expression alterations in IC genes across 537 ccRCC patient samples. Within cBioPortal, we utilised the Kidney Renal Clear Cell Carcinoma (TCGA, GDC) case set to study gene alterations in IC genes. ccRCC patients are represented as columns, and IC genes are represented as rows. Genomic alterations, including amplifications and deletions, alterations in gene expressions, and mutations are summarised by glyphs and colour coding. All cases are arranged as per alterations [35].

### 2.2. Bulk RNA-seq Data

RNA-sequencing transcriptomic data for 538 ccRCC tumours and 72 adjacent normal kidney tissue samples were obtained from the TCGA Kidney Renal Clear Cell Carcinoma (KIRC) STAR-aligned TPM dataset hosted by UCSC Xena (https://xenabrowser.net) (accessed on 10 February 2026) [36]. Gene expression values were quantified as transcripts per million (TPM) and were log2-transformed [log2(TPM + 1)] prior to downstream analyses. Differential gene expressions between normal kidney and ccRCC tumour samples were performed using GraphPad Prism (10.2.3 Software).

### 2.3. Prognostic Analysis

Cox proportional hazards regression analyses were performed using the SurvExpress online tool to evaluate the prognostic impact of immune checkpoint and EMT gene expression in 415 ccRCC patients [37]. For SurvExpress analyses, duplicated genes were averaged across all probe sets to generate a single expression value per sample, and the original quantile-normalised database was used. Combined EMT–IC prognostic models were constructed using multivariable Cox proportional hazards regression. Regression coefficients (β values) were estimated for each gene and were used to derive a prognostic index (risk score), defined as a linear combination of gene expression values weighted by their corresponding Cox coefficients (Risk score = Σβ × expression). Patients were ranked according to their prognostic index and stratified into high- and low-risk groups using the median risk score derived from the respective cohort distribution, generating two groups of comparable size. No alternative cut-point optimisation procedures (e.g., maximisation of risk separation) were applied. No formal adjustment for multiple hypothesis testing was applied, as analyses were exploratory in nature.

Kaplan–Meier survival curves were generated in RStudio (version 2025.9.2.418) to visualise survival differences between risk groups and validate the prognostic effect of individual genes or combined gene signatures. Hazard ratios (HRs) and 95% confidence intervals (CIs) comparing high- and low-risk groups were estimated by fitting a Cox proportional hazards model using risk group as the covariate. Model discrimination was assessed using the concordance index (C-index).

Forest plots were generated in RStudio to visualise the prognostic impact of individual immune checkpoints or combined EMT–IC gene signatures. Receiver operating characteristic (ROC) curves were also generated in SurvExpress to assess the discriminative performance of individual immune checkpoints and combined EMT–IC signatures for predicting overall survival. ROC curves were calculated by ranking patients based on gene-expression-derived risk scores from Cox models, and sensitivity and specificity were assessed across these rankings. Area under the curve (AUC) values with 95% confidence intervals quantified prognostic accuracy. Time-dependent predictive performance for LAG3 and NT5E was evaluated using AUC estimates across predefined time points (500–4000).

To determine whether immune checkpoint and EMT signatures provided prognostic information independent of established clinicopathological variables, multivariable Cox proportional hazards regression models were constructed in RStudio using the survival package. Separate models were fitted for NT5E, LAG3, the EMT signature score, NT5E + EMT, and LAG3 + EMT. All models were adjusted for age, sex, and pathological stage. HRs, 95% CIs, and Wald *p*-values were reported.

### 2.4. Single-Cell RNA Sequencing (scRNA-seq) Lineage Mapping and EMT Stratification

Publicly available single-cell transcriptomic count matrices from a cohort of ccRCC patients were obtained from the Gene Expression Omnibus (GEO) under accession number GSE210038. To map the cellular origins of immune checkpoint expression at single-cell resolution, raw counts from a representative patient sample (T5) were loaded and analysed using the Scanpy Python ecosystem.

#### 2.4.1. Preprocessing and Quality Control

Cells were filtered to retain only high-quality profiles expressing a minimum of 200 genes, and genes detected in fewer than three cells were excluded. To eliminate apoptotic cells and artefacts, downstream analysis was restricted to cells exhibiting less than 15% mitochondrial gene content. Total counts per cell were normalised to a target sum of 10,000 and log-transformed (log(1 + counts)). Following the selection of the top 3000 highly variable genes, data were scaled to a maximum variance threshold of 10.

#### 2.4.2. Dimensionality Reduction and Clustering

Principal Component Analysis (PCA) was performed on the scaled expression matrix using an ARPACK solver. A neighbourhood graph was constructed using the top 25 principal components with n neighbours = 15. Cells were embedded into low-dimensional space using Uniform Manifold Approximation and Projection (UMAP) and partitioned into transcriptionally distinct communities via the Leiden graph-clustering algorithm at a resolution of 0.5.

#### 2.4.3. Marker-Based Annotation and EMT State Classification

Leiden clusters were comprehensively annotated into major microenvironmental lineages using a pathology-validated dictionary of canonical ccRCC, stromal, and immune markers: Malignant/Tumour drivers: CA9, NDUFA4L2, SLC2A1, and EPCAM; Differentiated Epithelial: KRT8 and KRT18; Stromal/Fibroblasts: COL1A1, DCN, and PDG-FRA; Endothelial/Vascular: VWF, PTPRB, and EMCN; Immune Compartments: CD3D/CD3E (T-cells), NKG7/GNLY (NK-cells), LYZ/C1QA/FCGR3A (Myeloid), and HLA-DRA/CD74 (Antigen-Presenting Cells). Malignant cell clusters maintaining diagnostic ccRCC markers were further cross-referenced with pathway activity scores computed via AUCell to resolve their precise EMT state [38]. Tumour cells were stratified into Malignant_Epithelial-like (clusters retaining pristine epithelial/cytokeratin signatures but lacking structural fibroblast features) or a unified Malignant_EMT-like/Mesenchymal state (clusters co-expressing core ccRCC hypoxic drivers and structural mesenchymal/fibroblast collagens, or scoring above the cohort median for the HALLMARK_EPITHELIAL_MESENCHYMAL_TRANSITION signature) [39].

#### 2.4.4. Quantification of Checkpoint-Positive Proportions

Cells were defined as true positive for a given immune checkpoint if their log-normalised expression counts for LAG3 or NT5E were strictly greater than zero (>0). The proportional distribution of the checkpoint-positive compartment was calculated as a percentage fraction relative to the total number of positive cells detected across the entire microenvironment:

Proportional Fraction (%) = (Number of positive cells within Lineage i)/(Total number of positive cells in sample) × 100.

Data was compiled into distribution matrices and visualised using horizontal stacked bar charts to quantify the exact microenvironmental and tumour-intrinsic composition of checkpoint activity.

### 2.5. Correlation Analysis

Correlation analyses between EMT markers and IC genes were performed using bulk RNA-seq data from 535 ccRCC patients obtained from TCGA (GDC) via cBioPortal. Gene expression values were combined and normalised as FPKM Z-scores. Pearson and Spearman correlation coefficients were calculated in RStudio, and correlation tables were generated to assess the relationships between EMT and IC genes.

### 2.6. Immunohistochemistry Analysis

Immunohistochemistry was performed to assess co-localisation of LAG3 or NT5E with N-Cad or E-Cad using previously established protocols [40]. Paraffin-embedded tissue sections were deparaffinised, rehydrated, and subjected to antigen retrieval (pH 9.0 for N-Cad/E-Cad). Endogenous peroxidase activity was quenched with Dako peroxidase blocking solution, and non-specific binding was blocked with goat casein. Primary N-Cad antibody (Abcam, Cambridge, UK, ab98952; 1:1500) was applied overnight at 4 °C, followed by Dako, Glostrup, Denmark, Rabbit/Mouse secondary antibody (ENV K5007) and TSATM-GFP (1:100) labelling. Slides were then reprocessed for antigen retrieval (pH 9.0 for LAG3; pH 6.0 for NT5E), followed by peroxidase and non-specific protein blocking. Sections were incubated overnight at 4 °C with NT5E (Cell Signalling Technology, Danvers, MA, USA, mAB #13160; 1:100) or LAG3 (Abcam ab209236; 1:10) antibodies, followed by secondary antibody incubation and TSATM-Cy5 (1:100) detection. Nuclei were counterstained with DAPI, and slides were mounted using ProLong^®^ Diamond antifade mountant. Imaging was performed using an EVOS™ FL Auto 2 system (Thermo Fisher Scientific, Waltham, MA, USA).

Quantitative image analysis was performed using FIJI/ImageJ 2.16.0/1.54p/Java 1.8.0 (National Institutes of Health, Bethesda, MD, USA). Individual fluorescence channels (Cy5, GFP and DAPI) were separated and analysed independently. Fluorescence intensity of the immune checkpoint marker (LAG3 or NT5E; red channel) was measured as mean fluorescence intensity (MFI) and normalised to the corresponding DAPI fluorescence intensity to account for differences in tissue cellularity between analysed images. In parallel, the positive staining area (%) for the immune checkpoint marker was quantified following automatic Huang2 thresholding, with identical thresholding parameters applied across all analysed images. Four independently acquired tumour imaging fields were analysed for each staining group (LAG3 + NCad, LAG3 + ECad, NT5E + NCad, and NT5E + Ecad), and all individual measurements are presented in the quantitative analyses.

### 2.7. Multiplex Quantification of Soluble Immune Checkpoint Proteins

Plasma levels of soluble IC proteins were quantified using the LEGENDplex™ Human Immune Checkpoint Panel 1 (10-plex), according to the manufacturer’s instructions (Revvity, Scoresby, VIC, Australia). This bead-based multiplex immunoassay enables simultaneous detection of multiple analytes using flow cytometry. Briefly, patient plasma samples were incubated with fluorescently labelled capture beads conjugated to antibodies specific to IC proteins. Following incubation with detection antibodies and streptavidin-phycoerythrin, samples were acquired on a flow cytometer. Data were analysed using LEGENDplex™ software to determine analyte concentrations based on standard curves.

### 2.8. Statistical Analysis

All statistical analyses were performed in GraphPad Prism (GraphPad 10.2.3 Software) and R (version 4.3.1) using RStudio (version 2025.9.2.418). Differences in gene expression between normal kidney tissue and ccRCC tumours were assessed using the Wilcoxon rank-sum test. A *p*-value < 0.05 was considered statistically significant. RStudio was used to compute Kaplan–Meier analyses, hazard ratios (HRs), and 95% confidence intervals from Cox proportional hazards regression models, with statistical significance assessed using the log-rank test. The concordance index (C-index) was computed to assess predictive performance. ROC curves were generated in SurvExpress to evaluate the discriminative ability of individual ICs and combined EMT–IC signatures, with area under the curve (AUC) values calculated to quantify prognostic accuracy. Forest plots depicting HRs were generated in RStudio. Pearson and Spearman correlation analyses were also performed in RStudio to assess relationships between EMT and IC genes. Statistical significance across all analyses was represented consistently as: *p* < 0.05 (*), *p* < 0.01 (**), and *p* < 0.001 (***). All tests were two-sided, and *p*-values < 0.05 were considered statistically significant.

## 3. Results

### 3.1. Dysregulation of IC Molecules in ccRCC Patients

To identify IC molecules linked with immune escape in ccRCC, we analysed a panel of 20 genes based on our previous studies demonstrating associations between immune-modulatory genes and overall survival and recurrence in other solid cancers [22,23]. This panel comprised both immune stimulatory and inhibitory genes, including Hepatitis A Virus Cellular Receptor 2 (HAVCR2 (TIM-3)), Cluster of Differentiation 28 (CD28), Tumour Necrosis Factor Receptor Superfamily Member 9 (TNFRSF9 (CD137)), Fas Ligand (FASLG (CD95L)), T-cell immunoreceptor with Ig and ITIM domains (TIGIT), Galectin-9 (LGALS9), Cluster of Differentiation 80 (CD80), Cluster of Differentiation 27 (CD27), CTLA4, Lymphocyte Activation Gene-3 (LAG3), Indoleamine 2,3-dioxygenase 1 (IDO-1), V-Set Immunoregulatory Receptor (VSIR), PDCD1 (PD-1), Cluster of Differentiation 276 (CD276 (B7-H3)), Ecto-5′-Nucleotidase (NT5E (CD73)), Tumour Necrosis Factor Receptor Superfamily Member 14 (TNFRSF14 (HVEM)), B- and T-Lymphocyte Attenuator (BTLA), PD-L1 (CD274), VTCN1 (B7-H4), and Tumour Necrosis Factor Receptor Superfamily Member 18 (TNFRSF18 (GITR)).

**Figure 1:**
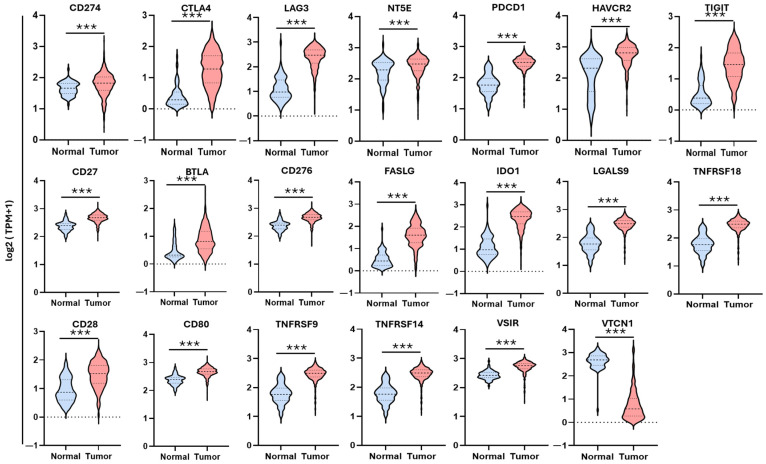
Violin plots illustrate the relative expression of 20 IC molecules in 72 adjacent normal kidney tissue samples (blue) and 538 ccRCC tumours (red). Gene expression values were log2-transformed [log2(TPM + 1)]. Statistical significance was assessed using the Wilcoxon rank-sum test. *** *p* < 0.001.

Differential expression of IC genes was evaluated using RNA-seq data from 538 ccRCC tumours and 72 adjacent normal kidney tissue samples obtained from the TCGA KIRC dataset. Comparative analysis revealed that the majority of IC genes analysed were significantly upregulated in ccRCC tissues relative to normal kidney tissues (*** *p* < 0.001), suggesting the presence of an immunosuppressive tumour microenvironment (Figure 1). Next, the expression profiles and genetic alterations of these 20 IC molecules in ccRCC patient tumours (*n* = 537) were assessed using OncoPrint analysis from cBioPortal. OncoPrint analysis revealed heterogeneous patterns of amplification, deletion, and mutation in several IC genes across the cohort (Supplementary Figure S1).

A bead-based multiplex LegendPlex immunoassay was performed to explore the modulation of IC proteins following immunotherapy with nivolumab. Plasma samples collected pre and post nivolumab treatment from a ccRCC patient were analysed. This patient had progressive disease, deterioration in general condition, and subsequently passed away. In plasma collected two weeks post-treatment, analysis demonstrated a reduction in soluble PD-L1 levels, accompanied by increased levels of multiple IC proteins, including PD-1, CTLA4, LAG3, Galectin-9 (LGALS9), TIM-3, CD27, CD25, and 4-1BB, suggesting systemic immune modulation following PD-1 blockade (Supplementary Figure S2). Overall, the observed dysregulation of multiple IC molecules in ccRCC highlights the potential utility of immunotherapeutic strategies targeting alternative or compensatory immune checkpoints in this cancer subtype.

### 3.2. ICs Prognosticate Poor Survival in ccRCC Patients

**Figure 2:**
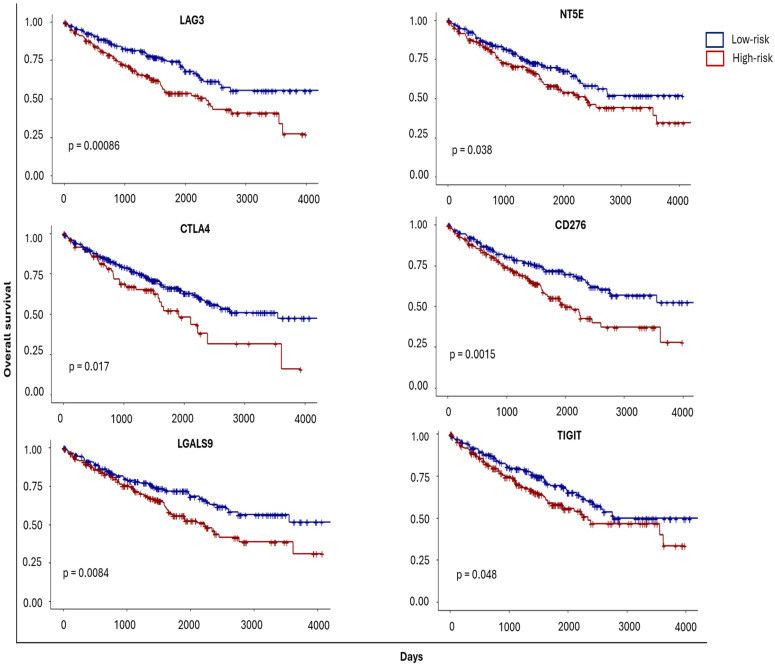
Association between inhibitory immune modulators and overall survival in ccRCC patients. Kaplan–Meier survival curves generated for the analysis of survival and gene expression of LAG3, NT5E, CTLA4, CD276, LGALS9, and TIGIT in 415 ccRCC patients. Blue curve represents low-risk group, while red curve represents high-risk group. The study time (days) is presented in the *x*-axis. Statistical significance was assessed using the log-rank test.

**Figure 3:**
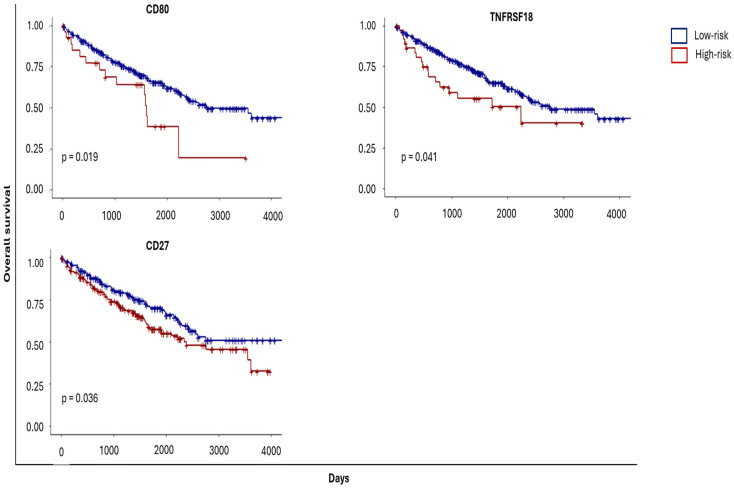
Association between stimulatory/co-stimulatory immune modulators and overall survival in ccRCC patients. Kaplan–Meier survival curves generated for the analysis of survival and gene expression of CD80, TNFRSF18, and CD27 in 415 ccRCC patients. Blue curve represents low-risk group, while red curve represents high-risk group. The study time (days) is presented in the *x*-axis. Statistical significance was assessed using the log-rank test.

To investigate the prognostic relevance of IC genes in ccRCC, we evaluated overall survival in a cohort of 415 ccRCC patients using SurvExpress. Kaplan–Meier survival risk curves for individual immune modulators were generated. Notably, altered expressions of immune inhibitory ICs, namely LAG3, NT5E, CTLA4, CD276, LGALS9, and TIGIT, were associated with significantly reduced overall survival (Figure 2). Altered expressions of stimulatory/co-stimulatory ICs such as CD80, TNFRSF18, and CD27 were linked with significantly decreased overall survival (Figure 3). In contrast, expression levels of other IC genes showed no significant correlation with overall survival (Supplementary Table S1). This finding suggests that individual ICs may contribute differently to patient outcomes and warrants further investigation to determine their biological and potential therapeutic relevance.

### 3.3. Association of IC Genes with EMT Markers Supports an EMT–IC Axis in ccRCC

**Figure 4:**
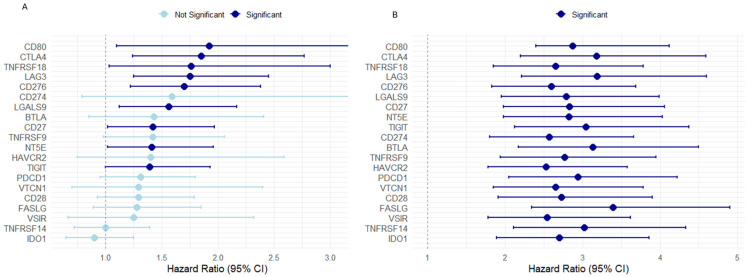
Prognostic impact of IC genes alone and in combination with EMT markers in 415 ccRCC patients. Forest plot showing Cox proportional hazards analysis of (**A**) 20 IC genes individually and (**B**) IC genes analysed in combination with EMT markers. Genes with a statistically significant association with overall survival are highlighted in dark blue, while non-significant genes are shown in light blue. Hazard ratios and 95% confidence intervals are shown.

To evaluate the prognostic significance of IC genes, Cox proportional hazards analysis was performed and visualised using forest plots. Among the 20 IC genes analysed, only a subset (*n* = 9) demonstrated a statistically significant association with overall survival in ccRCC patients (Figure 4A). Given the emerging role of EMT in regulating tumour immune evasion and IC expression, we evaluated the coordinated relationship between EMT markers and IC molecules to explore a potential EMT–IC axis in the same 415 ccRCC patient cohort. EMT markers E-cadherin (CDH1), N-cadherin (CDH2), Snail (SNAI1), Slug (SNAI2), Twist1, and Zeb1 were selected as they represent key components of the EMT programme, including loss of epithelial characteristics and activation of mesenchymal and transcriptional drivers of EMT. Notably, when combined with EMT gene expression, all IC genes exhibited a significant association with overall survival (Figure 4B). Furthermore, hazard ratios (HRs) for individual IC genes were generally modest (HR < 2). However, when analysed in combination with EMT markers, HR values increased substantially, with all genes exhibiting HRs greater than 2, indicating an enhanced prognostic stratification.

Multivariate Cox proportional hazards models were adjusted for clinical covariates including age, sex, and pathological stage and were used to assess independent prognostic effects. Neither LAG3, NT5E, nor the EMT score remained independently associated with overall survival. Additional models including EMT combined with NT5E or LAG3 similarly showed no independent prognostic significance for either variable after adjustment. In contrast, age and pathological stage remained strong independent predictors of outcome across all models (Supplementary Table S2). These results indicate that while LAG3 and NT5E are associated with survival in univariate analyses and contribute to combined EMT–IC risk stratification, their prognostic effects are not independent of standard clinicopathological variables in ccRCC.

**Figure 5:**
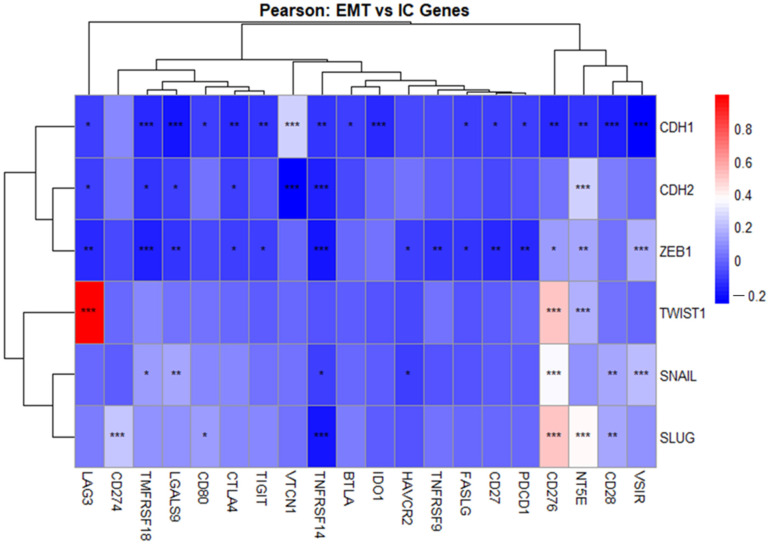
Correlation analysis of IC genes and EMT markers in ccRCC. Pearson correlation analyses were performed in 535 ccRCC patients between 20 IC genes and 6 EMT-related genes. In the heatmaps, red indicates positive correlation, blue indicates negative correlation, and the intensity reflects correlation strength; significance is indicated by *, **, and *** for *p* < 0.05, 0.01, and 0.001, respectively.

To further identify IC genes strongly associated with the EMT phenotype in ccRCC, correlation analyses were performed in 535 patients between 20 ICs and six EMT-related genes. Pearson correlation analysis revealed that most ICs and EMT-related genes were negatively correlated with CDH1, consistent with EMT activation, while correlations with CDH2 were generally weaker. LAG3 showed a robust positive correlation with TWIST1, CD276 correlated strongly with TWIST1 and SLUG and modestly with SNAIL, and NT5E showed a modest correlation with SLUG (Figure 5).

**Figure 6:**
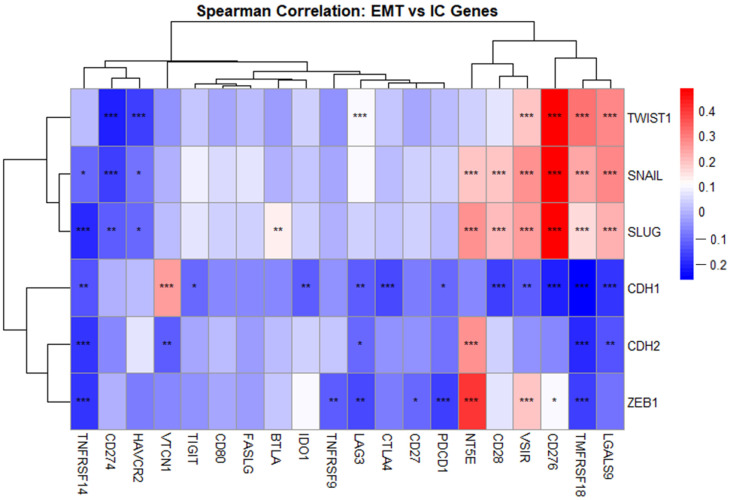
Correlation analysis of IC genes and EMT markers in ccRCC. Spearman correlation analyses were performed in 535 ccRCC patients between 20 IC genes and 6 EMT-related genes. In the heatmaps, red indicates positive correlation, blue indicates negative correlation, and the intensity reflects correlation strength; significance is indicated by *, **, and *** for *p* < 0.05, 0.01, and 0.001, respectively.

Spearman correlation analysis, which captures monotonic associations, confirmed negative correlations of most ICs with CDH1 and generally weak associations with CDH2, except for NT5E, which displayed strong positive correlations with CDH2, suggesting a link to mesenchymal features. Notably, LGALS9, TNFRSF18, and CD276 were positively associated with TWIST1, SNAIL, and SLUG, while CD28 correlated with SNAIL and SLUG. VSIR showed positive correlation with the four EMT transcription factors. NT5E showed strong positive correlations with CDH2, SNAIL, SLUG, and ZEB1. LAG3 also showed a positive association with TWIST1 (Figure 6).

Taken together, these analyses identified multiple immune checkpoint genes associated with EMT; however, LAG3 and NT5E were prioritised for further investigation based on consistent associations with EMT markers and survival outcomes, together with their representation of complementary immune regulatory axes, namely adaptive immune checkpoint signalling (LAG3) and purinergic/stromal–mesenchymal signalling (NT5E) [12]. LAG3 was further supported by its established clinical relevance as an immune checkpoint target, including FDA-approved therapeutic blockade in combination regimens, whereas NT5E was prioritised due to its strong association with mesenchymal markers and its demonstrated regulation within EMT-associated transcriptional programmes [30,41].

### 3.4. Co-Localisation of EMT and ICs in ccRCC Patients

**Figure 7:**
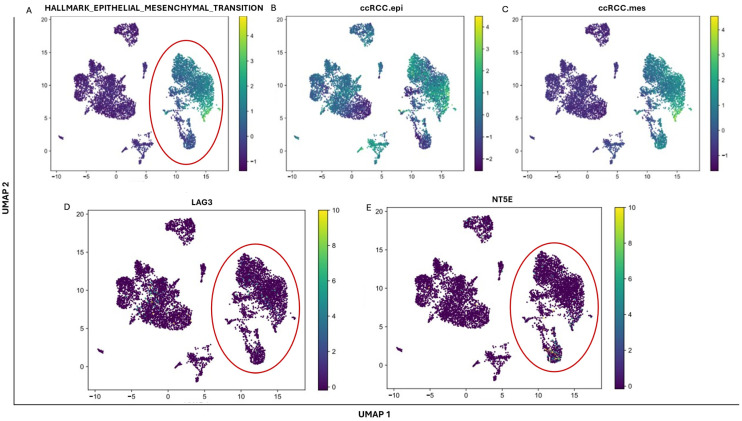
Single-cell co-localisation of EMT states and IC gene expression in ccRCC tumours. (**A**) UMAP plot showing EMT-positive tumour cells identified across ccRCC patients; a representative patient is shown. Red circle indicates EMT-positive cluster. (**B**) Distribution of epithelial tumour cells classified using the ccRCC.epi signature. (**C**) Distribution of mesenchymal tumour cells classified using the ccRCC.mes signature. UMAP plot showing expression of (**D**) LAG3 and (**E**) NT5E across tumour cells. Red circle highlights EMT-positive cluster co-expressing LAG3 or NT5E.

To evaluate the relationship between EMT status and two IC genes, LAG3 and NT5E, single-cell transcriptomic data from seven ccRCC patients were analysed. Tumour cells were classified using the ccRCC.epi and ccRCC.mes signatures to distinguish epithelial and mesenchymal states. UMAP plots illustrate EMT-positive cells (Figure 7A), with epithelial and mesenchymal populations highlighted separately (Figure 7B,C). Across all patient samples, tumour cells exhibited co-expression of EMT markers and IC genes LAG3 (Figure 7D) and NT5E (Figure 7E), indicating that immune-related programmes are active in both epithelial and mesenchymal tumour cells. A representative patient sample is presented in Figure 7, while a second patient sample displaying similar expression patterns is presented in Supplementary Figure S3.

Additionally, other IC genes were also observed to align with EMT-positive cells across patient samples (Supplementary Figure S4). Notably, the expression of IC genes was not restricted to tumour cells but was also detected in multiple non-tumour cell types within the TME. To determine the cellular origin of LAG3 and NT5E expression, single-cell transcriptomic data from ccRCC patients were subjected to cell-type-resolved lineage tracking (Supplementary Figure S5 and Table S3). Rather than exhibiting uniform tumour-intrinsic expression, both checkpoints demonstrated clear compartmentalisation across the TME. Purely epithelial-like malignant cells accounted for a negligible fraction of checkpoint expression (0.43% of LAG3+ and 1.78% of NT5E cells). Within the malignant compartment, IC expression was predominantly associated with transitional EMT-like states. Malignant EMT-like/mesenchymal tumour cells accounted for 31.74% of all LAG3-positive cells and 8.28% of all NT5E-positive cells, indicating preferential enrichment of checkpoint activity in phenotypically plastic tumour states. In addition, a substantial proportion of LAG3 expression was attributable to infiltrating T-cell and lymphoid populations (40.87%), while NT5E expression was largely associated with endothelial and vascular cells (51.48%) (Table S3). Together, these findings demonstrate that IC gene expression in ccRCC is distributed across both tumour and stromal compartments, but is preferentially enriched in EMT-like malignant cells. This supports the presence of an EMT–IC axis, in which checkpoint activity is linked to tumour cell plasticity as well as a remodelled TME.

**Figure 8:**
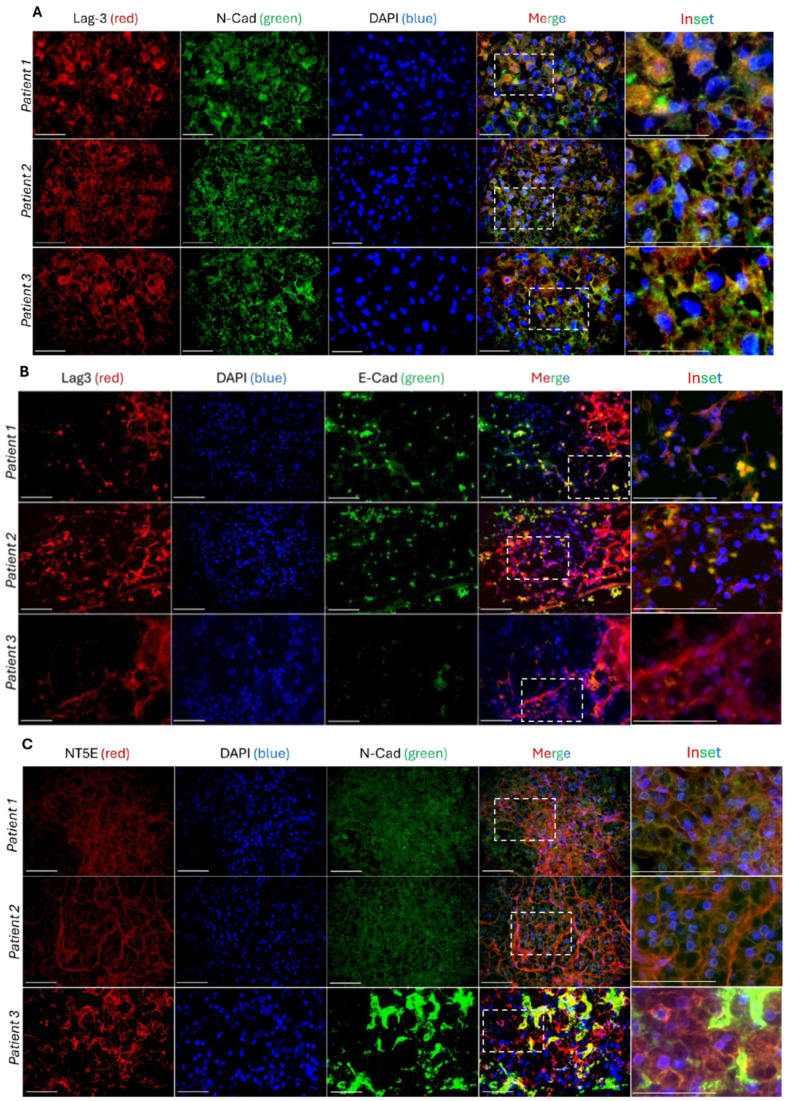

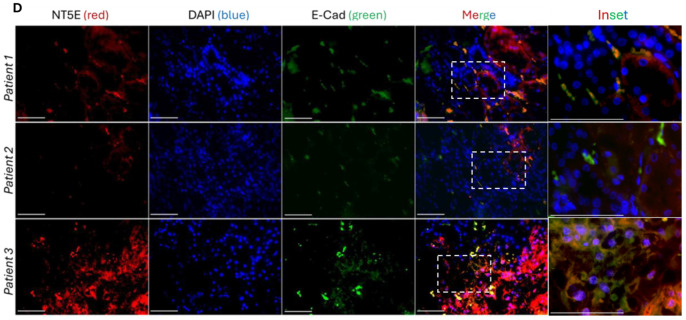
Localisation of LAG3 with (**A**) mesenchymal and (**B**) epithelial markers; NT5E with (**C**) mesenchymal and (**D**) epithelial markers in ccRCC tumour tissues. Representative immunohistochemical staining of LAG3 or NT5E (red) in combination with the mesenchymal marker N-cad (green) and the epithelial marker E-cad (green) in three ccRCC tumour samples. Nuclei were counterstained with DAPI (blue). Merged images show the overlay of all three channels, along with inset (white dash box). Scale bar 100 µm.

Next, we performed immunohistochemistry to examine the localisation of the epithelial marker E-cadherin and the mesenchymal marker N-cadherin with LAG3 and NT5E (Figure 8). Across the three patient samples analysed, LAG3 and NT5E expression were observed in subsets of both E-cadherin-positive and N-cadherin-positive tumour cells. Quantitative image analysis of normalised fluorescence intensity and positive staining area further confirmed immune checkpoint expression within both epithelial- and mesenchymal-associated tumour regions (Supplementary Figure S6). These findings indicate the co-expression of LAG3 and NT5E with both epithelial and mesenchymal markers within the tumour, further supporting the presence of an EMT–IC axis in ccRCC.

### 3.5. Time-Dependent ROC Analysis of the EMT–IC Axis in ccRCC

**Figure 9:**
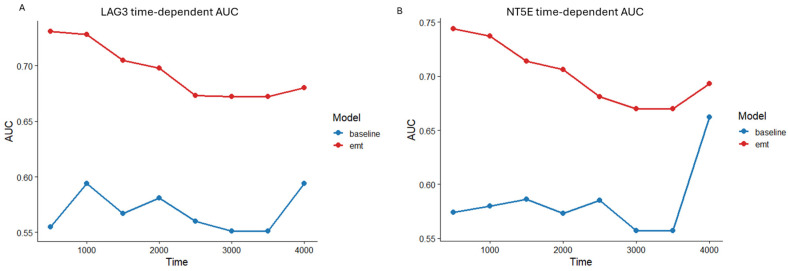
Time-dependent AUC analysis of IC genes alone (baseline) and in combination with EMT markers for prediction of overall survival in 415 ccRCC patients. Time-dependent AUCs showing the predictive performance of (**A**) LAG3 alone and the combined LAG3–EMT model, (**B**) NT5E alone, and the combined NT5E–EMT model across time points ranging from 500 to 4000 days.

ROC analysis was performed to evaluate the prognostic performance of IC genes alone and in combination with EMT markers for overall survival in 415 ccRCC patients (Supplementary Figure S7). Individually, both IC genes LAG3 and NT5E showed limited prognostic ability, with AUC values remaining relatively stable over time (Figure 9). Individually, LAG3 showed AUC values remaining relatively stable over time, ranging from approximately 0.55 at 500 days to 0.59 at 4000 days. In contrast, combining LAG3 with the six EMT markers moderately improved prognostic performance. The combined model achieved an AUC of 0.73 at 500 days to 0.68 at 4000 days (Figure 9A). Similarly, NT5E alone showed AUC values ranging from 0.57 at 500 days to 0.66 at 4000 days. The combined EMT–NT5E model showed improved prognostic accuracy overall, with AUC values ranging from 0.73 at 500 days to 0.68 at 4000 days (Figure 9B). The combined models consistently outperformed IC genes alone across all time points, with the strongest prognostic performance observed at earlier time points. Although the AUCs indicate moderate predictive accuracy, these findings support the biological relevance of the EMT–IC axis in ccRCC rather than immediate clinical application.

## 4. Discussion

This study provides a multi-platform analysis of the relationship between EMT-associated transcriptional programmes and immune checkpoint expression in ccRCC, identifying a prognostically relevant EMT–IC axis involving LAG3 and NT5E. Across bulk transcriptomic, single-cell, and immunohistochemical analyses, immune checkpoints were consistently associated with EMT-related cellular states, supporting a biologically coordinated link between tumour plasticity and immune regulation. Both genes were associated with poor overall survival in univariate analyses, and their integration with the EMT phenotype improved risk stratification compared with individual immune checkpoint markers. Although NT5E and LAG3 were not independent prognostic factors in multivariable analysis, integration of EMT features improved their time-dependent discriminatory performance, highlighting the potential value of combining EMT and immune checkpoint information.

ccRCC is a highly heterogeneous tumour, and robust biomarkers are urgently needed to improve prognostic determination and inform treatment decisions. Our findings, together with those of previous findings, indicate IC expression and pathway dysregulation as prominent features of ccRCC [33,42]. Multiple immune checkpoint genes, including CTLA4, CD80, TNFRSF18, CD276, LGALS9, CD27, and TIGIT, were associated with poor overall survival in ccRCC, supporting widespread dysregulation of immune regulatory pathways in this tumour type [43,44,45,46,47,48,49]. Our analysis of 537 ccRCC patients versus 72 normal kidney samples revealed significant upregulation of 19 out of 20 ICs in ccRCC patient tumours. In contrast, a previous study which examined 534 patients and 15 IC genes overlapped with five of the ICs we interrogated, namely PD-L1, CTLA4, LAG3, HAVCR2, and CD276 [33]. PD-L1 showed discordant results between the studies, where PD-L1 differential expression did not reach significance in the previous study [33]. This discrepancy may reflect the small increase in patient numbers and differences in data preprocessing and analysis methods. Although PD-L1 was upregulated in ccRCC, it showed no significant association with patient outcomes in this cohort, consistent with some but not all previous studies [25,50]. This discrepancy may reflect tumour microenvironmental heterogeneity [51].

EMT is increasingly recognised as a driver of invasion and metastasis that also re-models the TME by promoting immunosuppressive signalling, recruitment of suppressive immune populations, and reduced anti-tumour immune activity, thereby facilitating immune evasion [52,53]. Our findings support this concept by demonstrating coordinated expression of immune checkpoint molecules with EMT-associated transcriptional programmes in ccRCC. Correlation analyses, single-cell RNA sequencing, and immunohistochemistry consistently showed enrichment of LAG3 and NT5E within EMT-associated tumour populations, supporting the existence of an EMT–IC axis. While multiple immune checkpoint genes showed prognostic associations, integrative analyses suggest that LAG3 and NT5E represent central components of this EMT-associated immune regulatory programme, indicating that specific immune checkpoints may be more closely linked to tumour plasticity and patient outcomes.

Lymphocyte-activation gene 3 (LAG3 or CD223) negatively regulates T-cell activation and function, and its blockade enhances effector T-cell activity, with multiple inhibitors currently under evaluation in ccRCC clinical trials [54]. In this study, LAG3 expression was associated with poor overall survival, consistent with previous reports, and aligns with single-cell studies demonstrating enrichment in dysfunctional T-cell populations in ccRCC. Collectively, these findings support a link between LAG3-associated immune dysfunction and EMT-associated tumour plasticity within the ccRCC tumour microenvironment.

NT5E (CD73) is a glycophosphatidylinositol-anchored ectoenzyme that generates adenosine to suppress T-cell activation [20,23]. NT5E expression was associated with higher tumour grades and poor overall survival in ccRCC, consistent with previous studies in both ccRCC and other solid tumours [22,23,44]. Together with prior evidence of EMT-dependent regulation of NT5E, these findings further support a mechanistic link between tumour plasticity and adenosine-mediated immune suppression. Overall, these observations suggest that EMT-associated tumour states may contribute to immune evasion through coordinated activation of immune regulatory pathways, with LAG3 and NT5E representing components of this EMT-associated immune programme rather than isolated molecular events.

Both LAG3 and NT5E were not independently associated with overall survival after adjustment for clinical covariates. These findings indicate that EMT-associated and IC-related transcriptional signals are not independent prognostic determinants in ccRCC, and may, at least in part, reflect underlying clinical disease characteristics captured by standard prognostic factors such as tumour stage and patient age. Our integrated analyses demonstrate a coordinated association between IC genes and EMT-related transcriptional programmes, supporting the existence of a biologically linked EMT–IC axis in ccRCC. However, this axis does not appear to provide prognostic information beyond established clinical variables, suggesting that its signal is largely embedded within broader tumour progression biology rather than functioning as an independent prognostic driver.

A strength of this study is the integration of complementary transcriptomic approaches, with prognostic analyses based on established canonical EMT markers and independent single-cell analyses using ccRCC epithelial and mesenchymal gene signatures to investigate EMT-associated cellular states. While the six-gene EMT panel captures key regulators of EMT, it does not encompass the full spectrum of EMT-associated transcriptional programmes, which are increasingly recognised to exist as dynamic partial and hybrid epithelial–mesenchymal states. A study reported that EMT signatures correlated closely with IC gene signatures and could influence clinical outcomes in ccRCC patients [33]. N-cad was the only EMT marker in our study that overlapped with the 23 core EMT genes evaluated [33]. Another study suggested that ccRCC patients with a mesenchymal status determined by the expression of E-cad, N-cad, Vimentin, and Fibronectin may respond better to combination treatment with ICIs and anti-angiogenic therapy [34]. We have previously demonstrated that EMT and ICs bidirectionally influence each other to facilitate tumour aggressiveness [30,52,55]. Future studies using broader EMT transcriptional signatures and functional approaches will further refine the relationship between EMT plasticity and immune checkpoint regulation in ccRCC.

Furthermore, an increase in secreted LAG3 levels in a ccRCC patient with poor clinical response to PD-1 blockade supports the role of LAG3 as a compensatory IC. Although based on a single exploratory case, this observation raises the possibility that LAG3 may have potential as both a predictive biomarker of ICI resistance and a candidate target for combination immunotherapeutic strategies in ccRCC, particularly within EMT-like tumour cell populations. These findings should be interpreted cautiously and require validation in larger, clinically annotated cohorts. These findings align with prior single-cell studies demonstrating a highly dysfunctional immune microenvironment in ccRCC characterised by exhausted and CXCL13^+^ CD8^+^ T-cell states [56], supporting the concept that immune dysregulation and tumour cell plasticity represent interconnected processes.

A limitation of this study is that ICI therapy analyses were restricted to a single exploratory ccRCC case, precluding conclusions regarding therapy resistance or the predictive value of LAG3. These findings are therefore hypothesis-generating, and validation in larger, clinically annotated ccRCC cohorts treated with ICIs will be required to determine their predictive relevance. Prospective validation will also be important to confirm the clinical relevance of the proposed EMT–IC axis. In addition, key clinical variables such as tumour grade, metastatic status, and treatment information were not consistently available in the TCGA-KIRC cohort and could not be included in multivariable analyses; thus, the independence of the EMT–IC axis should be interpreted in the context of available covariates (age, sex, and pathological stage). The single-cell RNA sequencing and immunohistochemistry datasets were also limited in sample size and should be regarded as complementary, hypothesis-supporting evidence providing cellular context for the transcriptomic findings. Further validation in larger independent cohorts is required to confirm the cell-type-specific distribution of immune checkpoints and their association with EMT states. Functional studies, including EMT induction models in ccRCC cell lines, will be important to determine whether EMT directly regulates LAG3, NT5E, and related immune checkpoints, thereby providing mechanistic validation of the EMT–IC axis.

Finally, no formal correction for multiple testing was applied in exploratory gene-level analyses; therefore, these results should be interpreted within a hypothesis-generating framework. Future work incorporating external validation, calibration analysis, and comparison with established clinicopathological prognostic models will be necessary to determine the clinical utility of the EMT–IC axis in ccRCC.

Collectively, our findings support a framework in which EMT contributes to the establishment of an immunosuppressive TME through coordinated IC activation. Targeting EMT-associated immune regulatory pathways, including LAG3- and NT5E-mediated signalling, may therefore represent a rational strategy to overcome immune evasion and improve response to ICIs in ccRCC.

## 5. Conclusions

ccRCC is the most lethal type of urogenital tumour, with an increasing mortality rate over several years. Accordingly, better therapies with durable responses are needed to treat ccRCC patients. ICI-based immunotherapies alone or in combination with TKIs show promise for the non-surgical treatment of ccRCC. In this study, we investigated the relationship between immune checkpoint expression and EMT status in ccRCC, identifying an EMT–IC axis in which EMT-like tumour cell states are associated with NT5E and LAG3 expression. This axis was associated with adverse prognosis and improved risk stratification when integrated with EMT features, suggesting potential relevance for prognostic assessment and therapeutic targeting. These findings support further investigation of LAG3 and NT5E as candidate biomarkers within EMT-associated tumour states in ccRCC.

## Data Availability

The data presented in this study are available upon request from the corresponding authors.

## Supplementary Materials

The following supporting information can be downloaded at: https://www.mdpi.com/article/10.3390/cancers18142258/s1, Figure S1. The OncoPrint analysis showing alterations in gene expression of immune checkpoint molecules in ccRCC patients. Rows and columns depict genes and ccRCC patients, respectively. Genomic alterations, such as deletions and amplifications, mutations, and changes in expression of genes are summarised by glyphs and color coding. The cases are represented as per alterations. Figure S2. Multiplex analysis of soluble immune checkpoint proteins in plasma from a ccRCC patient before and after nivolumab treatment. Figure S3. Additional representative single-cell co-localisation of EMT states and LAG3 or NT5E gene expression in ccRCC tumours. (A) UMAP plot showing EMT-positive tumour cells identified across an additional representative patient is shown. Red circle indicates EMT-positive cluster (B) Distribution of epithelial tumour cells classified using the ccRCC.epi signature. (C) Distribution of mesenchymal tumour cells classified using the ccRCC.mes signature. UMAP plot showing expression of (D) LAG3 and (E) NT5E across tumour cells. Red circle highlighted EMT-positive cluster co-expressing LAG3 or NT5E. Figure S4. Single-cell co-localisation of EMT states and immune checkpoint gene expression in ccRCC tumours. UMAP plot showing expression of (A) PDCD1, (B) CD274, (C) CTLA4, (D) CD276, (E) HAVCR2 and (F) TNFRSF18 across tumour cells. Red circle highlighted EMT-positive cluster. Figure S5. Cell type annotation and checkpoint-positive cell distribution in a representative ccRCC patient. (A) UMAP visualisation of Leiden clusters identified from single-cell RNA sequencing data of a patient. Clusters were annotated based on canonical lineage marker expression and classified as malignant epithelial-like tumour cells (cluster 11), malignant EMT-like/mesenchymal tumour cells (clusters 0 and 3), T-cell/lymphoid cells (clusters 1 and 2), NK-like/cytotoxic cells (clusters 4 and 9), myeloid/APC populations (clusters 7, 8, 10 and 12), endothelial cells (cluster 6) or fibroblasts (cluster 5). (B) Dot plot showing the expression of canonical lineage markers across Leiden clusters used for cell type annotation. Dot size represents the proportion of cells expressing a given marker, while colour intensity reflects average expression. (C) Stacked bar plot showing the proportional distribution of NT5E-positive and LAG3-positive cells across annotated cellular compartments. Figure S6. Quantitative image analysis of (A) normalised fluorescence intensity and (B) positive staining area confirming immune checkpoint expression within both epithelial- and mesenchymal-associated tumour regions. Four independently acquired tumour imaging fields analysed for each staining group (LAG3+NCad, LAG3+ECad, NT5E+NCad and NT5E+Ecad). Figure S7. Time-dependent ROC analysis of IC genes alone and in combination with EMT markers for prediction of overall survival in ccRCC patients. Time-dependent ROC curves showing the predictive performance of (A) LAG3 alone, (B) the combined LAG3–EMT model, (C) NT5E alone, and (D) the combined NT5E–EMT model across time points ranging from 500 to 4000 days. Table S1. SurvExpress-based overall survival of 415 ccRCC patients. Table S2. Multivariate Cox proportional hazard regression analyses in 412 ccRCC patients. Hazard ratios (HRs) with 95% confidence intervals are shown. EMT score represents the first principal component derived from the six EMT markers (CDH1, CDH2, SNAI1, SNAI2, TWIST1 and ZEB1). Table S3. Distribution of LAG3- and NT5E-expressing cells across major cell populations identified by single-cell RNA sequencing in ccRCC.

## Abbreviations

The following abbreviations are used in this manuscript:

AUC	Area under the curve
B7-H3	B7 homologue 3
BTLA	B- and T-Lymphocyte Attenuator
C-index	Concordance index
ccRCC	Clear cell renal cell carcinoma
CD	Cluster of Differentiation
CTLA4	Cytotoxic T-lymphocyte-associated antigen 4
EMT	Epithelial-to-mesenchymal transition
FASLG	Fas Ligand
GITR	Glucocorticoid-induced tumour necrosis factor family receptor
HAVCR2	Hepatitis A Virus Cellular Receptor 2
HRs	Hazard ratios
ICs	Immune checkpoints
ICIs	Immune checkpoint inhibitors
IDO-1	Indoleamine 2,3-dioxygenase 1
KIRC	Kidney Renal Clear Cell Carcinoma
LAG3	Lymphocyte Activation Gene-3
mTOR	Mammalian target of rapamycin
NT5E	Ecto-5′-Nucleotidase
ORR	Overall response rate
PD-1	Programmed cell-Death 1
PD-L1	Programmed Death Ligand 1
RCC	Renal cell carcinoma
ROC	Receiver operating characteristic
scRNA-seq	Single-cell RNA sequencing
TIGIT	T-cell immunoreceptor with Ig and ITIM domains
TKIs	Tyrosine kinase inhibitors
TME	Tumour microenvironment
TNFRSF	Tumour Necrosis Factor Receptor Superfamily Member
TPM	Transcripts per million
UMAP	Uniform Manifold Approximation and Projection
VEGF	Vascular endothelial growth factor
VHL	Von Hippel Lindau
VSIR	V-Set Immunoregulatory Receptor
