# Supplementary material for: Interplay Between Immune Checkpoint Modulators and the Epithelial-to-Mesenchymal Transition Axis in Clear Cell Renal Cell Carcinoma
Source: Cancers (Basel). 2026 Jul 14;18(14):2258. doi: 10.3390/cancers18142258 (PMC13406277; doi:10.3390/cancers18142258)
Supplement: Supplementary file 1 [file cancers-18-02258-s001.zip › cancers-4316276-supplementary.pdf]

cancers

MDPI

Article

Interplay Between Immune Checkpoint Modulators and the Epithelial-to-Mesenchymal Transition Axis in Clear Cell Renal Cell Carcinoma

Arpita Poddar [1,2,3,4,†], Farah Ahmady-Nield [1,2,†], Revati Sharma [1], Seemadri Subhadarshini [5,6], Mohit Kumar Jolly [5], Suresh Ramakrishna [7,8], Ali Raza [1], Ravi Shukla [3,9,10], George Kannourakis [1,2], Aparna Jayachandran [1,2,*] and Prashanth Prithviraj [1,2,*]

1 Fiona Elsey Cancer Research Institute, Ballarat, VIC 3350, Australia; arpita@fecri.org.au (A.P.); farah@fecri.org.au (F.A.-N.); revupattani@gmail.com (R.S.); alir335612@gmail.com (A.R.); george@fecri.org.au (G.K.)
2 School of Institute of Innovation, Science and Sustainability, Federation University, Ballarat, VIC 3350, Australia
3 Ian Potter NanoBiosensing Facility, NanoBiotechnology Research Laboratory, School of Science, RMIT University, Melbourne, VIC 3000, Australia; ravi.shukla@rmit.edu.au
4 Department of Surgery, The Royal Melbourne Hospital, The University of Melbourne, Parkville, VIC 3050, Australia
5 Department of Bioengineering, Indian Institute of Science, Bengaluru 560012, Karnataka, India; seemadri.subhadarshini@well.ox.ac.uk (S.S.); mkjolly@iisc.ac.in (M.K.J.)
6 Nuffield Department of Medicine, University of Oxford, Oxford OX3 7BN, UK
7 Graduate School of Biomedical Science and Engineering, Hanyang University, Seoul 04763, Republic of Korea; suri28@hanyang.ac.kr
8 College of Medicine, Hanyang University, Seoul 04763, Republic of Korea
9 Centre for Advanced Materials and Industrial Chemistry, RMIT University, Melbourne, VIC 3001, Australia
10 School of Health & Biomedical Science, RMIT University, Melbourne, VIC 3000, Australia
* Correspondence: aparna@fecri.org.au (A.J.); prashanth@fecri.org.au (P.P.)
† These authors contributed equally to this work.

 https://doi.org/10.3390/cancers18142258

Supplementary Data

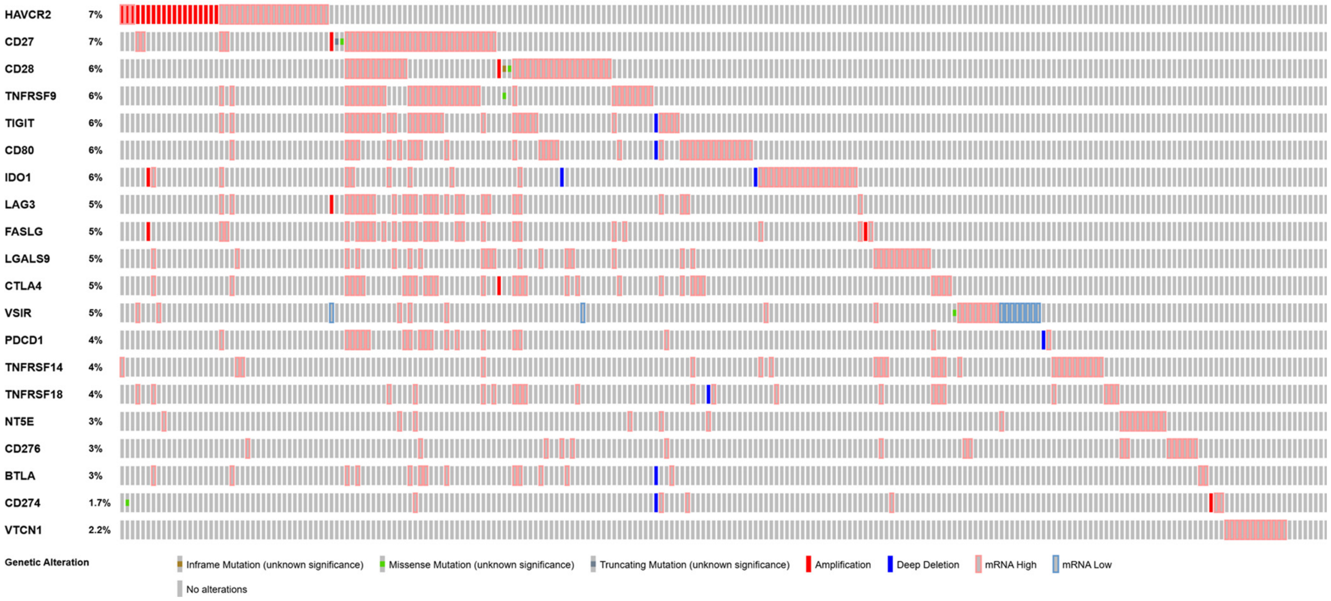

Supplementary Figure S1. The OncoPrint analysis showing alterations in gene expression of immune checkpoint molecules in ccRCC patients.

Rows and columns depict genes and ccRCC patients, respectively. Genomic alterations, such as deletions and amplifications, mutations, and changes in expression of genes are summarised by glyphs and color coding. The cases are represented as per alterations.

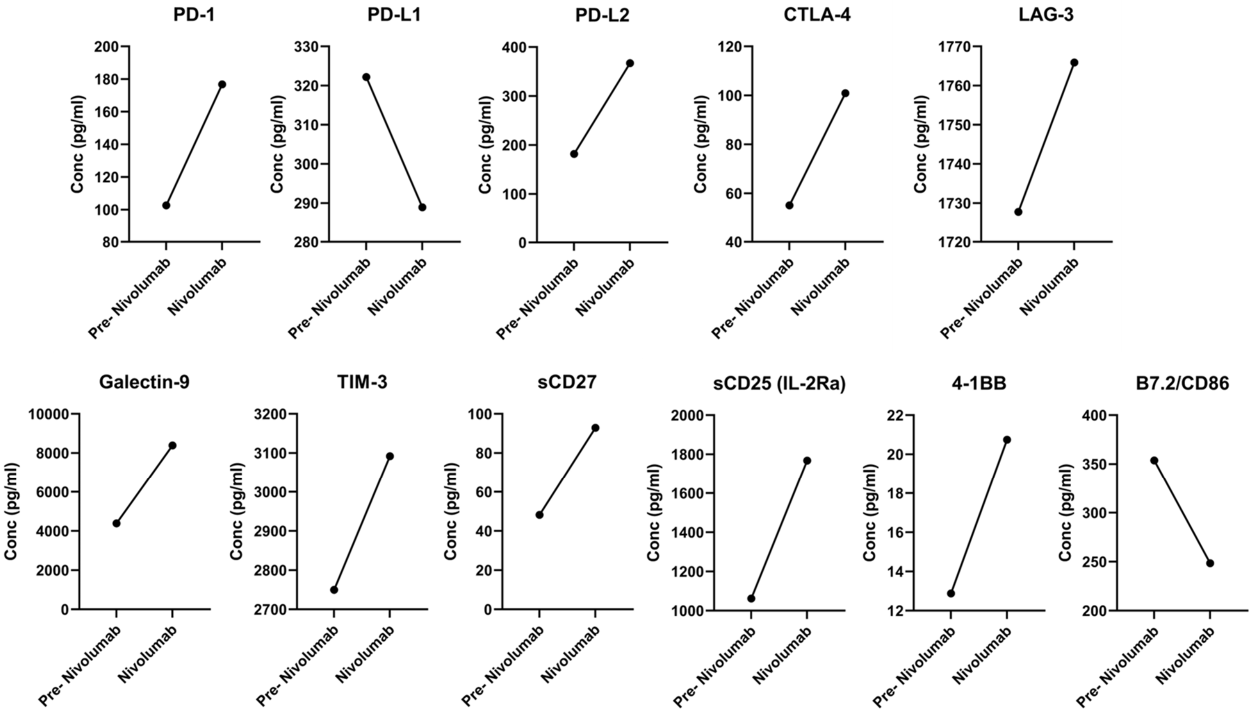

Supplementary Figure S2. Multiplex analysis of soluble immune checkpoint proteins in plasma from a ccRCC patient before and after nivolumab treatment.

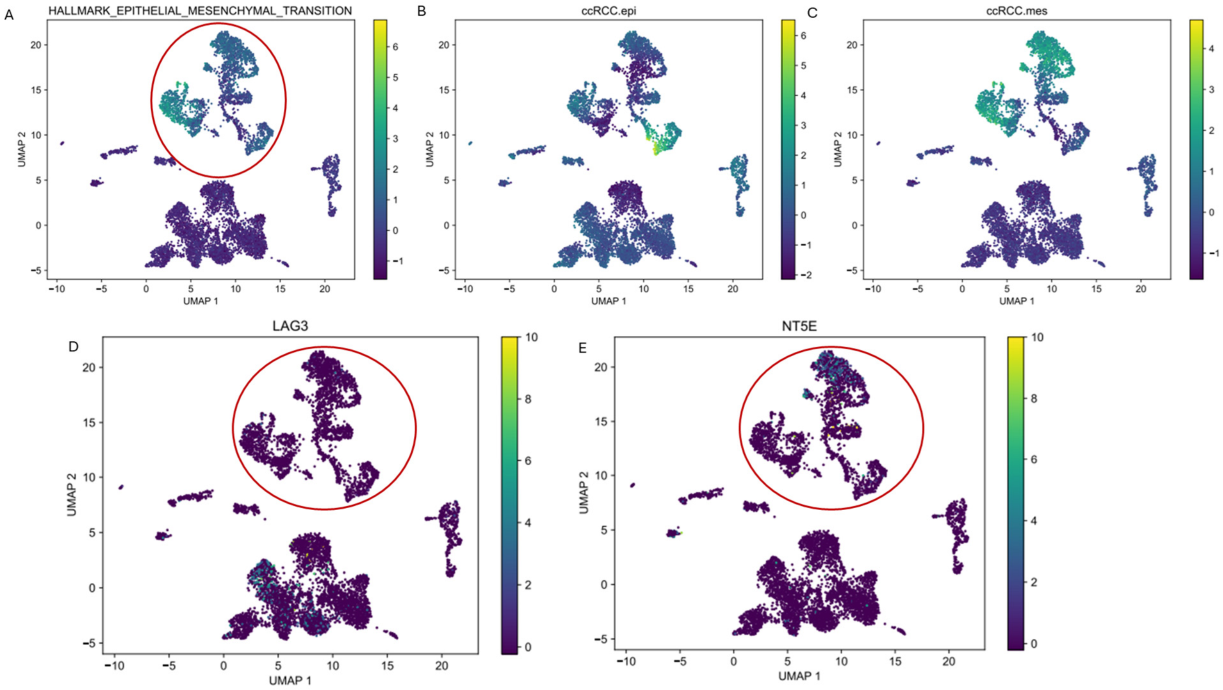

Supplementary Figure S3. Additional representative single-cell co-localisation of EMT states and LAG3 or NT5E gene expression in ccRCC tumours.

(**A**) UMAP plot showing EMT-positive tumour cells identified across an additional representative patient is shown. Red circle indicates EMT-positive cluster (**B**) Distribution of epithelial tumour cells classified using the ccRCC.epi signature. (**C**) Distribution of mesenchymal tumour cells classified using the ccRCC.mes signature. UMAP plot showing expression of (**D**) LAG3 and (**E**) NT5E across tumour cells. Red circle highlighted EMT-positive cluster co-expressing LAG3 or NT5E.

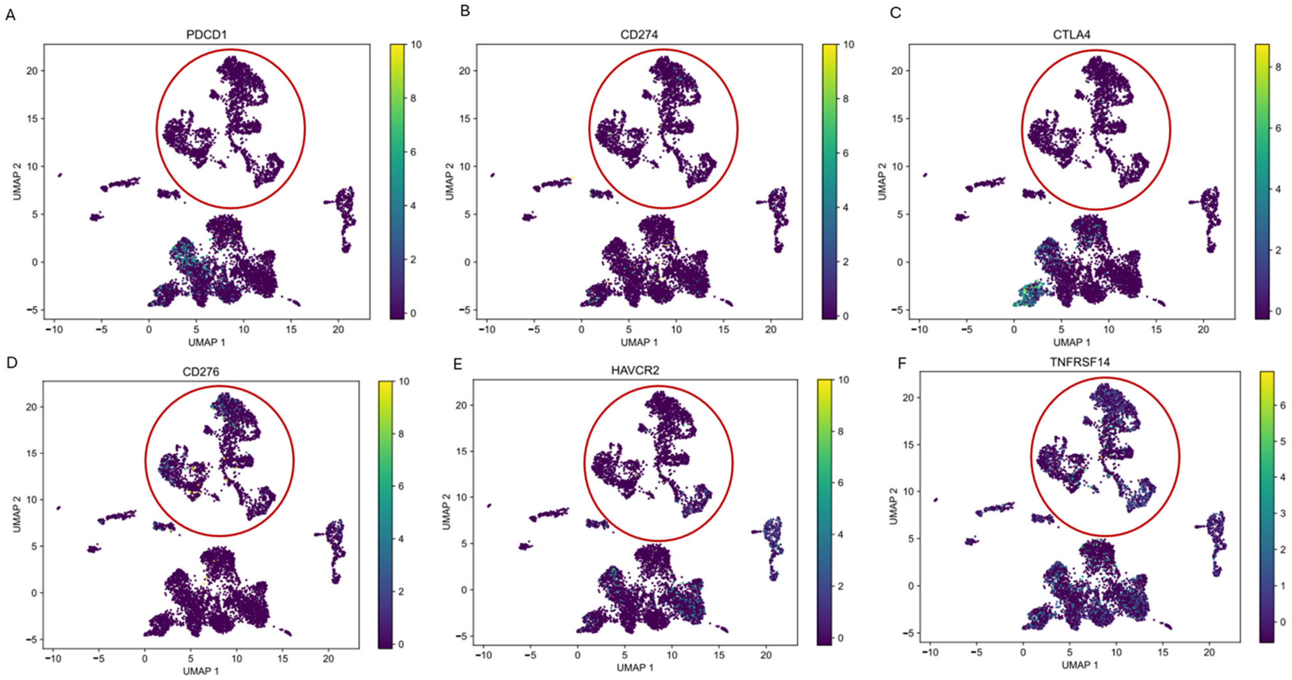

Supplementary Figure S4. Single-cell co-localisation of EMT states and immune checkpoint gene expression in ccRCC tumours.

UMAP plot showing expression of (**A**) PDCD1, (**B**) CD274, (**C**) CTLA4, (D) CD276, (**E**) HAVCR2 and (**F**) TNFRSF18 across tumour cells. Red circle highlighted EMT-positive cluster.

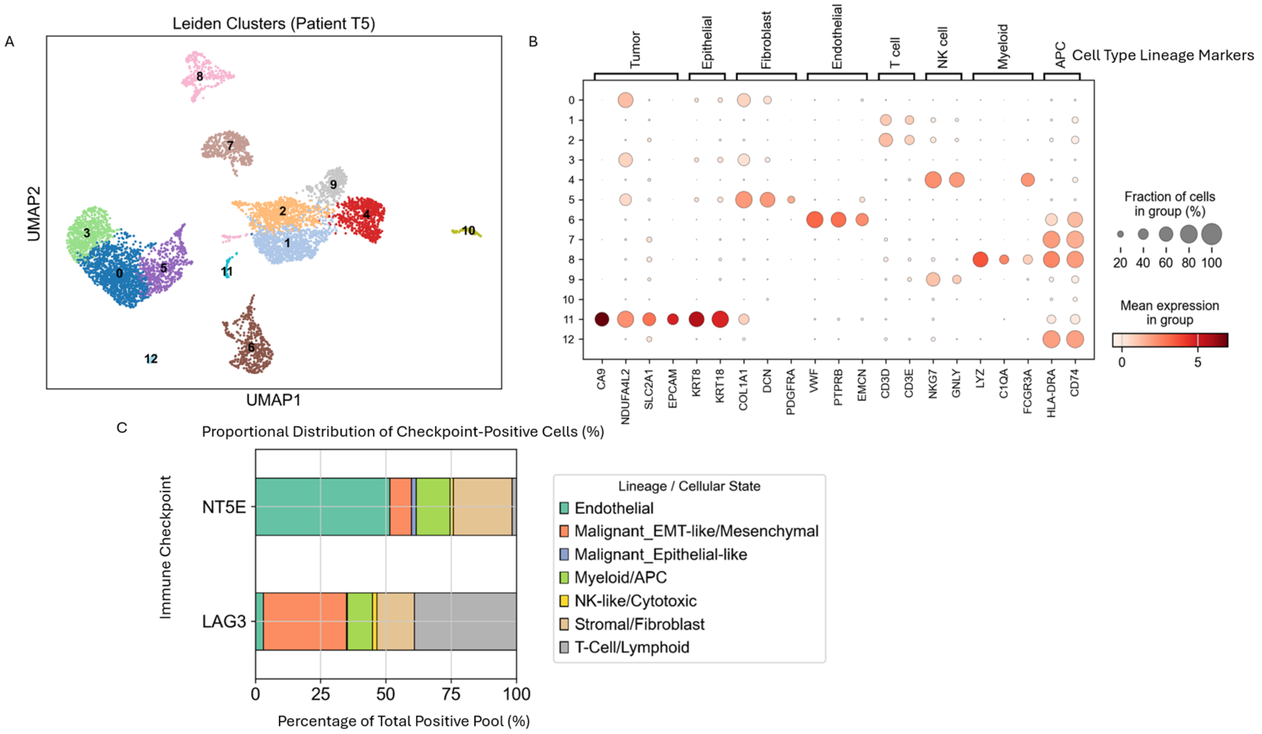

Supplementary Figure S5. Cell type annotation and checkpoint-positive cell distribution in a representative ccRCC patient.

(**A**) UMAP visualisation of Leiden clusters identified from single-cell RNA sequencing data of a patient. Clusters were annotated based on canonical lineage marker expression and classified as malignant epithelial-like tumour cells (cluster 11), malignant EMT-like/mesenchymal tumour cells (clusters 0 and 3), T-cell/lymphoid cells (clusters 1 and 2), NK-like/cytotoxic cells (clusters 4 and 9), myeloid/APC populations (clusters 7, 8, 10 and 12), endothelial cells (cluster 6) or fibroblasts (cluster 5). (**B**) Dot plot showing the expression of canonical lineage markers across Leiden clusters used for cell type annotation. Dot size represents the proportion of cells expressing a given marker, while colour intensity reflects average expression. (**C**) Stacked bar plot showing the proportional distribution of NT5E-positive and LAG3-positive cells across annotated cellular compartments.

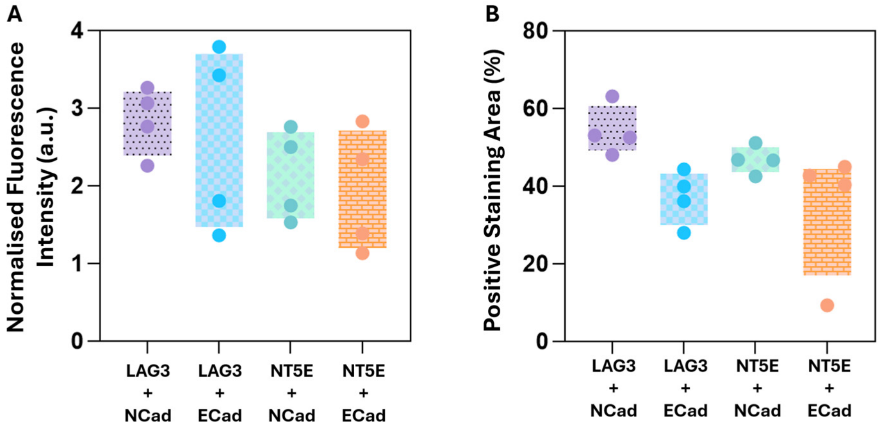

Supplementary Figure S6. Quantitative image analysis of (**A**) normalised fluorescence intensity and (**B**) positive staining area confirming immune checkpoint expression within both epithelial- and mesenchymal-associated tumour regions.

Four independently acquired tumour imaging fields analysed for each staining group (LAG3+NCad, LAG3+ECad, NT5E+NCad and NT5E+Ecad).

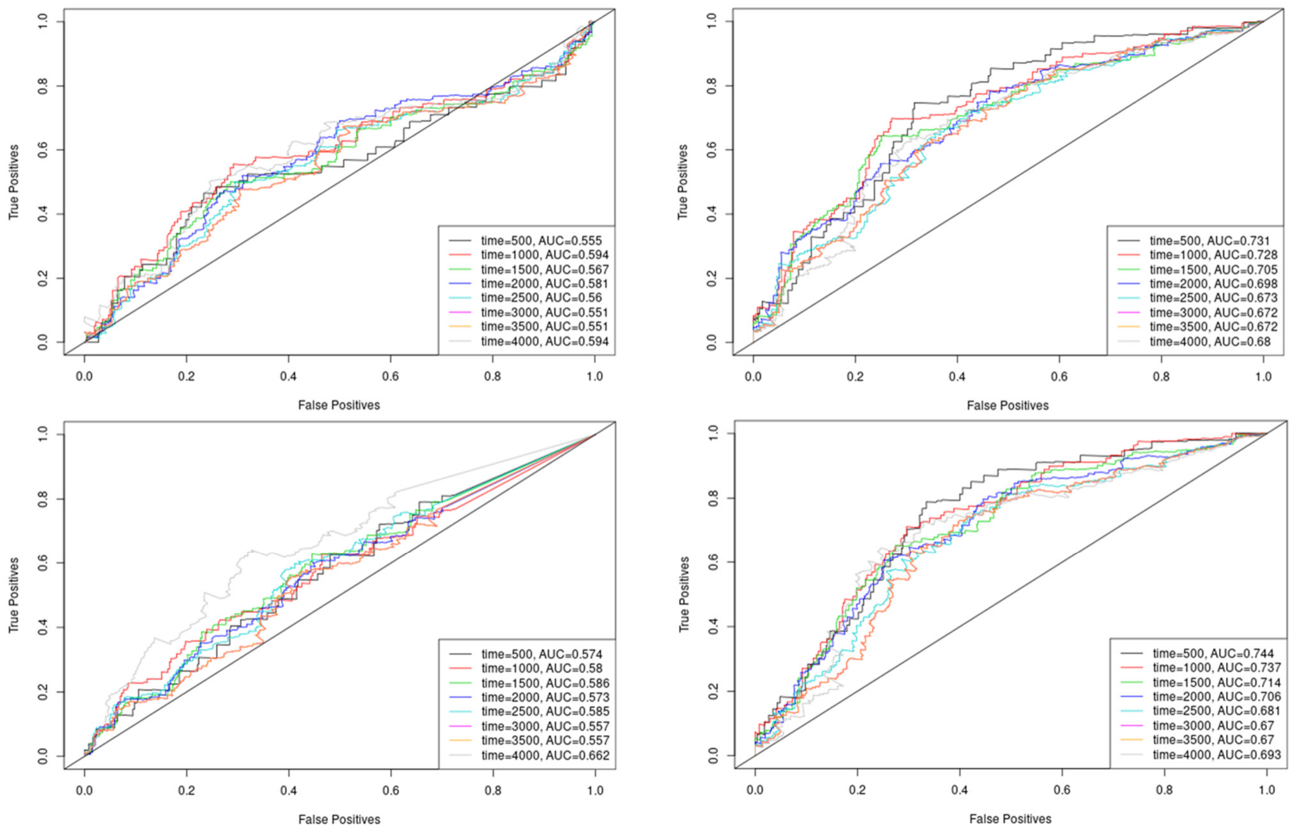

Supplementary Figure S7. Time-dependent ROC analysis of IC genes alone and in combination with EMT markers for prediction of overall survival in ccRCC patients.

Time-dependent ROC curves showing the predictive performance of (**A**) LAG3 alone, (**B**) the combined LAG3–EMT model, (**C**) NT5E alone, and (**D**) the combined NT5E–EMT model across time points ranging from 500 to 4000 days.

Table S1. SurvExpress-based overall survival of 415 ccRCC patients.

Immune-Modulatory Gene	Risk groups Hazard Ratio	Confidence Interval	Log-Rank Equal Curves (*p* value)
HAVCR2	1.4	0.75 ~ 2.59	0.289
CD28	1.29	0.93 ~ 1.79	0.128
TNFRSF9	1.42	0.98 ~ 2.06	0.061
FASLG	1.28	0.89 ~ 1.85	0.188
IDO1	0.9	0.65 ~ 1.25	0.535
VSIR	1.25	0.67 ~ 2.32	0.477
TNFRSF14	1	0.72 ~ 1.39	0.980
BTLA	1.43	0.85 ~ 2.41	0.179
CD274	1.59	0.79 ~ 3.17	0.190
VTCN1	1.29	0.7 ~ 2.4	0.416
PDCD1	1.31	0.95 ~ 1.8	0.104

Table S2. Multivariate Cox proportional hazard regression analyses in 412 ccRCC patients.

Hazard ratios (HRs) with 95% confidence intervals are shown. EMT score represents the first principal component derived from the six EMT markers (CDH1, CDH2, SNAI1, SNAI2, TWIST1 and ZEB1).

Variables	Clinical Covariates	NT5E	LAG3	EMT	NT5E+ EMT	LAG3+ EMT
**NT5E**	—	0.95 (0.80–1.12), *p* = 0.547	—	—	0.93 (0.77–1.11), *p* = 0.406	—
**LAG3**	—	—	0.98 (0.82–1.18), *p* = 0.831	—	—	0.99 (0.82–1.18), *p* = 0.877
**EMT score**	—	—	—	0.97 (0.87–1.08), *p* = 0.577	0.95 (0.84–1.07), *p* = 0.402	0.97 (0.87–1.09), *p* = 0.591
**Age**	1.04 (1.02–1.06), *p* < 0.001	1.04 (1.02–1.06), *p* < 0.001	1.04 (1.02–1.06), *p* < 0.001	1.04 (1.02–1.06), *p* < 0.001	1.04 (1.02–1.06), *p* < 0.001	1.04 (1.02–1.06), *p* < 0.001
**Sex**	0.96 (0.68–1.35), *p* = 0.82	0.96 (0.68–1.35), *p* = 0.80	0.96 (0.69–1.36), *p* = 0.83	0.97 (0.69–1.37), *p* = 0.88	0.97 (0.69–1.37), *p* = 0.88	0.98 (0.69–1.37), *p* = 0.88
**Stage II**	1.05 (0.54–2.07), *p* = 0.87	1.05 (0.54–2.06), *p* = 0.88	1.06 (0.54–2.07), *p* = 0.87	1.06 (0.54–2.08), *p* = 0.86	1.06 (0.54–2.07), *p* = 0.86	1.07 (0.54–2.08), *p* = 0.85
**Stage III**	2.22 (1.44–3.41), *p* < 0.001	2.23 (1.45–3.40), *p* < 0.001	2.22 (1.44–3.41), *p* < 0.001	2.21 (1.44–3.40), *p* < 0.001	2.22 (1.45–3.42), *p* < 0.001	2.21 (1.44–3.39), *p* < 0.001
**Stage IV**	5.77 (3.81–8.73), *p* < 0.001	5.84 (3.86–8.86), *p* < 0.001	5.79 (3.82–8.77), *p* < 0.001	5.72 (3.78–8.66), *p* < 0.001	5.81 (3.83–8.80), *p* < 0.001	5.73 (3.78–8.69), *p* < 0.001

Table S3. Distribution of LAG3- and NT5E-expressing cells across major cell populations identified by single-cell RNA sequencing in ccRCC.

Cell Type Lineage / State	Proportional Distribution of LAG3+ cells (%)	Proportional Distribution of NT5E+ cells (%)
Lymphoid Populations (T-Cell / NK)	40.87	2.96
Myeloid / APC Components	19.57	13.02
Stromal / Fibroblast Components	14.35	22.49
Endothelial / Vascular Components	3.04	51.48
Malig-nant_Epithelial-like (Pure Tumor)	0.43	1.78
Malignant_EMT-like / Mesenchymal	31.74	8.28

https://doi.org/10.3390/cancers18142258
